# Editorial: E3 ubiquitin ligases: critical roles in cancer progression and therapeutic implications

**DOI:** 10.3389/fonc.2026.1995835

**Published:** 2026-10-01

**Authors:** Praveen Koganti, Pengda Liu

**Affiliations:** 1 Cancer Genome and Epigenetics Program, Sanford Burnham Prebys Medical Discovery Institute, La Jolla, CA, United States; 2 Lineberger Comprehensive Cancer Center, University of North Carolina at Chapel Hill, Chapel Hill, NC, United States; 3 Department of Biochemistry and Biophysics, University of North Carolina at Chapel Hill, Chapel Hill, NC, United States

**Keywords:** deubiquitinase, E3 ubiquitin ligase, protein substrates, therapy response, tumorigenesis

Protein homeostasis is a critical hallmark-enabling process in cancer (1) and plays a central role in tumorigenesis (2), tumor progression, and therapeutic responses (3). Among the major mechanisms governing protein homeostasis, ubiquitin-dependent protein degradation provides a highly dynamic system for regulating the stability and activity of target proteins (4). Protein ubiquitylation is mediated through a sequential E1/E2/E3 enzymatic cascade, in which E3 ubiquitin ligases confer substrate specificity (5), whereas deubiquitinases (DUBs) remove ubiquitin modifications and thereby regulate protein stability and signaling (6). Given the diverse and context-dependent substrates controlled by individual E3 ligases and DUBs, these enzymes can exert highly cancer- and tissue-specific functions. By harnessing the protein degradation or stabilization activities of E3 ligases and DUBs, respectively, PROTACs (proteolysis-targeting chimera) (7) and DUBTACs (deubiquitinase-targeting chimera) (8) have emerged as novel therapeutic strategies, translating our understanding of the ubiquitin system into clinical applications.

The rapid advancement of bioinformatic approaches, together with the increasing availability of multi-omics and functional profiling datasets across tumor types, has enabled systematic investigation of ubiquitin-regulated pathways in cancer. These studies are not only revealing the roles of specific ubiquitin-related enzymes in individual cancer contexts, but also establishing connections between ubiquitin-dependent regulation, tumor phenotypes, therapeutic responses, and clinical outcomes (9). Such work has highlighted the potential of ubiquitin pathway components as prognostic biomarkers, predictive markers, and therapeutic targets. This collection brings together 4 original research articles, 1 review article, and 1 Opinion article that highlight several emerging frontiers in ubiquitin biology and its implications for cancer.

Gonga-Cave et al. investigated the C-terminal to LisH (CTLH) complex, a multi-subunit E3 ubiquitin ligase with context-dependent functions in cancer. By generating mice with combined deletion of Ranbp9, a core component of the CTLH complex, and p53, the authors found accelerated sarcoma development compared with either single deletion, supporting a tumor-suppressive role for the CTLH complex in sarcomagenesis.

Zang et al. identified RARS1, a protein synthesis-associated factor, as a key regulator of ENO1 protein stability in liver hepatocellular carcinoma (LIHC). RARS1 suppresses ENO1 ubiquitylation and degradation, thereby promoting tumor progression and resistance to ferroptosis. This study illustrates how cancer cells can exploit the ubiquitin-dependent protein degradation machinery to stabilize oncogenic proteins and promote both tumorigenesis and therapeutic resistance.

Xu et al. performed a pan-cancer analysis of the E3 ubiquitin ligase PELI1 and further investigated its function in LIHC. Their analyses and experimental validation revealed an oncogenic role for PELI1 through regulation of both tumor-intrinsic cellular programs and the tumor immune microenvironment. Collectively, these findings highlight the diagnostic, prognostic, and immunomodulatory potential of PELI1 and support its further investigation as a therapeutic target in LIHC.

In esophageal squamous cell carcinoma (ESCC), Lin et al. systematically analyzed ubiquitination- and deubiquitination-related genes and developed an 11-gene signature with high predictive accuracy for patient prognosis. Functional validation of NEURL3 further supported the biological relevance of this signature. This study demonstrates the potential of integrating ubiquitin pathway profiling with bioinformatic and experimental approaches to improve prognostic stratification and identify therapeutic opportunities in ESCC.

Jiang et al. provided a comprehensive review of current knowledge regarding E3 ubiquitin ligases and deubiquitinases in renal cell carcinoma, with a particular focus on their molecular mechanisms and emerging opportunities for therapeutic targeting. The review highlights the increasingly important roles of ubiquitin-dependent regulation in renal cancer biology and treatment.

Finally, in an Opinion article, Barik et al. discussed an intriguing recently reported function of ABLIM1 as an E3 ubiquitin ligase that targets IκBα for ubiquitylation and degradation, thereby regulating NF-κB signaling in colorectal cancer. This emerging finding expands our understanding of the functional diversity of ubiquitin ligases and illustrates how previously unrecognized ubiquitin-dependent mechanisms can contribute to cancer signaling and progression.

Together, these studies underscore the diverse roles of ubiquitin-mediated protein regulation in cancer and its dependence on cellular and tumor context. They also demonstrate the value of combining mechanistic studies with computational and clinical analyses to better understand ubiquitin signaling and to identify potential biomarkers and therapeutic targets.
